# Continuous Catheter Versus Single-Shot Interscalene Block in Shoulder Surgery: A Review and Recommendations for Postoperative Pain Management

**DOI:** 10.7759/cureus.75332

**Published:** 2024-12-08

**Authors:** Thomas L Karadimas, Kalie L Nuss, Ryan D Bridgeport, Morgan James, Panteli Hapipis, Austin Buchanan, James Champane

**Affiliations:** 1 Medicine, Morsani College of Medicine, USF Health, Tampa, USA; 2 Natural Science, College of Natural Science, Michigan State University, East Lansing, USA; 3 Emergency Medicine, Henry Ford Health System, Detroit, USA

**Keywords:** continuous interscalene block, opioid reduction, pain management, shoulder surgery, single-shot interscalene block

## Abstract

Shoulder arthroscopic and arthroplastic surgeries are associated with significant postoperative pain, which can delay recovery and increase opioid consumption. Interscalene blocks (ISBs) are a commonly used method to manage this pain, either as single-shot injections or continuous catheter infusions (CISBs). This review synthesizes findings from studies conducted in the past five years, comparing the efficacy, complications, and outcomes of single-shot ISBs versus CISBs for postoperative pain management in shoulder surgeries. Current literature highlights key differences: single-shot ISBs provide significant immediate postoperative pain relief, whereas CISBs offer prolonged analgesia beyond 48 hours, reduced opioid consumption, and enhanced recovery outcomes. However, CISBs carry a higher risk of complications and procedural complexity compared to single-shot ISBs. Both single-shot ISBs and CISBs present effective options for postoperative pain control in shoulder surgery patients. Single-shot ISBs may be preferable for patients seeking immediate pain relief with fewer complications, while CISBs are beneficial for those requiring prolonged analgesia. The choice of technique should be individualized based on the patient’s needs, expected recovery, and potential risk factors.

## Introduction and background

Over 500 thousand shoulder arthroscopies and 100 thousand shoulder arthroplasties are performed each year, highlighting the substantial volume of shoulder surgeries conducted annually. The burden of shoulder surgery is expected to continue increasing due to the aging population and the need for revision [1,2]. The prevalence of postoperative pain in orthopedic surgery is significant, with up to 80% of patients reporting moderate to severe pain within the first 48 hours [3-6]. Postoperative pain can lead to delayed rehabilitation, decreased mobility, and increased opioid consumption, necessitating the use of safe and effective local management of pain to ensure positive outcomes for patients undergoing such procedures. Despite heightened attention in recent decades, recent reports highlight the ongoing issue of inadequate treatment for acute postoperative pain, especially in patients status post-shoulder surgery [7,8].

There are multiple ways to approach postoperative pain in shoulder surgery, including preemptive medication, local anesthetics, and regional anesthetics, as well as oral medications such as acetaminophen, NSAIDs, tramadol, and gabapentin. Another frequently employed regional anesthesia technique to deal with this ubiquitous and debilitating postoperative pain is an interscalene block (ISB), which is used in over 40% of shoulder arthroplasty cases and shows superiority in pain relief compared to local anesthetic injections alone [9-13]. These blocks involve the injection of local anesthetics specifically around the major nerves comprising the brachial plexus, which supply both motor and sensory functions of the shoulder [14]. ISBs can be administered either as a single injection or through a continuous interscalene catheter block (CISB) with a patient-controlled analgesia (PCA) pump.

There is a continued discussion on which ISB approach is superior, and despite the recent advancements in technique and implants in shoulder arthroscopy and arthroplasty, there remains a paucity of updated recommendations concerning the regional management of postoperative pain, namely in the choice of ISBs [15]. Thus, with the increasing burden of shoulder surgery and the subsequent pain that ensues, it is paramount that clinicians are given updated recommendations to approach postoperative pain with ISBs. This review will discuss the mechanisms, effectiveness, complications, and overall recommendations for single-shot ISB versus CISB for postoperative pain for patients undergoing shoulder surgery.

## Review

ISB overview

Brachial Plexus Function and Anatomy

The brachial plexus is a key network of nerves that transmits signals responsible for motor and sensory innervation of the upper extremities, including the shoulder, arm, and hand. It originates from the ventral rami of the C5 to T1 spinal nerves [16]. The brachial plexus provides all motor and most sensory functions to the shoulder, with the exception being the supraclavicular nerve, arising from the superficial cervical plexus (C3-C4), which is responsible for innervating the dermatome on the superior aspect of the shoulder [17].

The first major nerve of the brachial plexus involved in shoulder innervation relevant to shoulder surgery pain management is the suprascapular nerve, which originates from the C5 and C6 nerve roots of the superior trunk of the brachial plexus. The suprascapular nerve provides motor innervation to the supraspinatus and infraspinatus muscles, which are two of the four muscles that make up the rotator cuff. It provides sensory innervation to most of the shoulder, including the coracohumeral ligament, coracoclavicular ligament, subacromial bursa, posterior shoulder capsule, acromioclavicular (AC) joint, glenohumeral joint, and dermatome on the posterior aspect of the shoulder [18]. The second major nerve of the brachial plexus involved in an ISB is the axillary nerve, which also originates from the C5 and C6 nerve roots, but instead from the posterior cord of the brachial plexus. The axillary nerve provides motor innervation to the deltoid and teres minor muscles and sensory innervation to the skin overlying the deltoid muscle [19].

**Figure 1:**
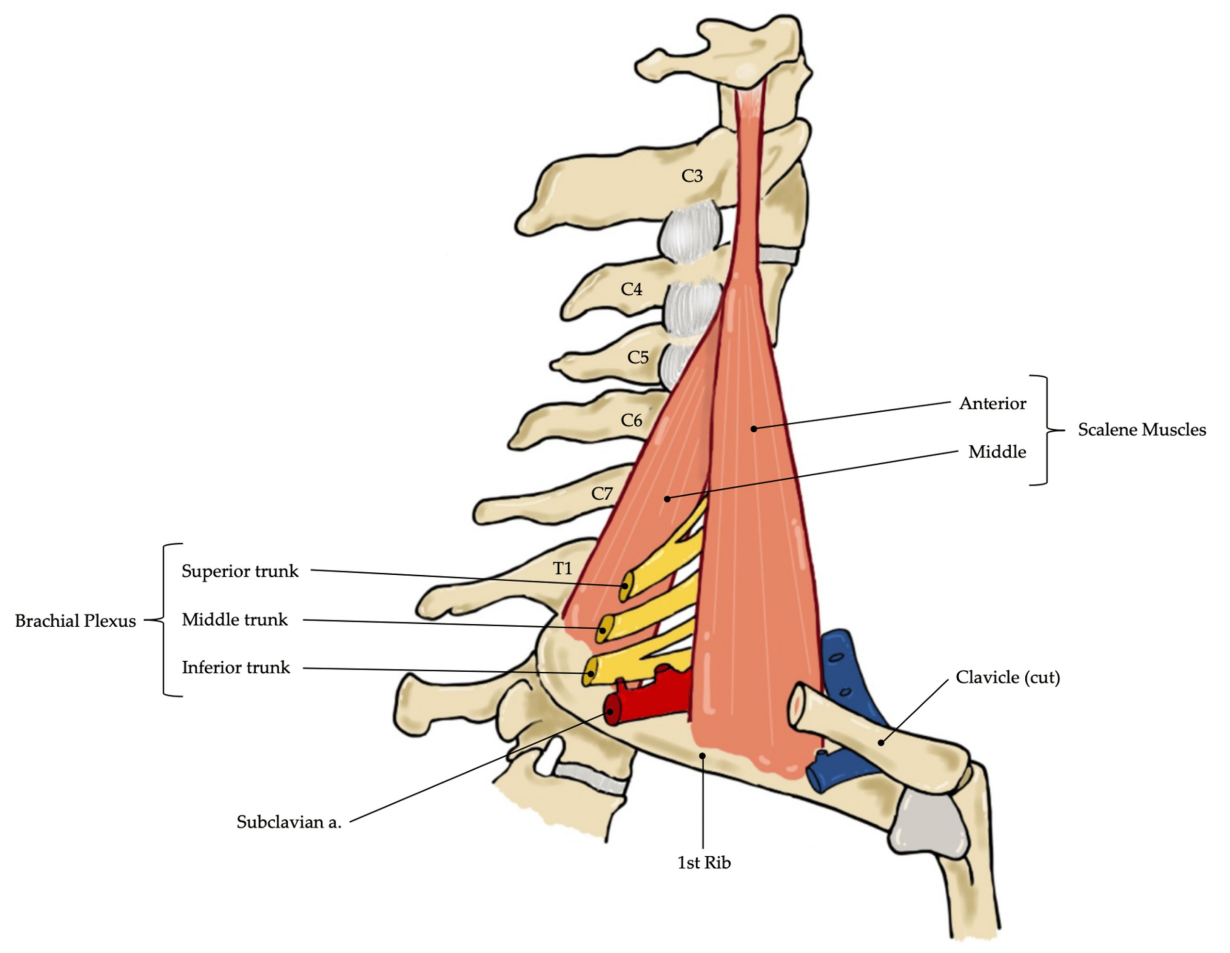
Brachial Plexus Anatomy The C5 to T1 nerve roots pass between the anterior and middle scalene muscles. The superior, middle, and inferior trunks of the brachial plexus are formed as these nerve roots descend through the interscalene groove. Image credit: Kalie L. Nuss via iArtbook

As the nerve roots of the brachial plexus converge to form trunks, they travel through the interscalene groove (ISG) between the anterior and middle scalene muscles at the level of the 6th cervical vertebra, which is approximately aligned with the cricoid cartilage (CC) located medially [20]. The anterior and middle scalene muscles lie deep to the sternocleidomastoid (SCM) muscle, originating from the transverse processes of the cervical vertebrae and inserting into distinct aspects of the first rib. The space between them, known as the interscalene groove (ISG), provides a pathway for the nerve roots to travel distally (Figure 1). These nerve roots are positioned superior and posterior to the subclavian artery and give rise to the superior (C5 and C6), middle (C7), and inferior (C8 and T1) trunks of the brachial plexus [21].

ISB Administration

**Figure 2:**
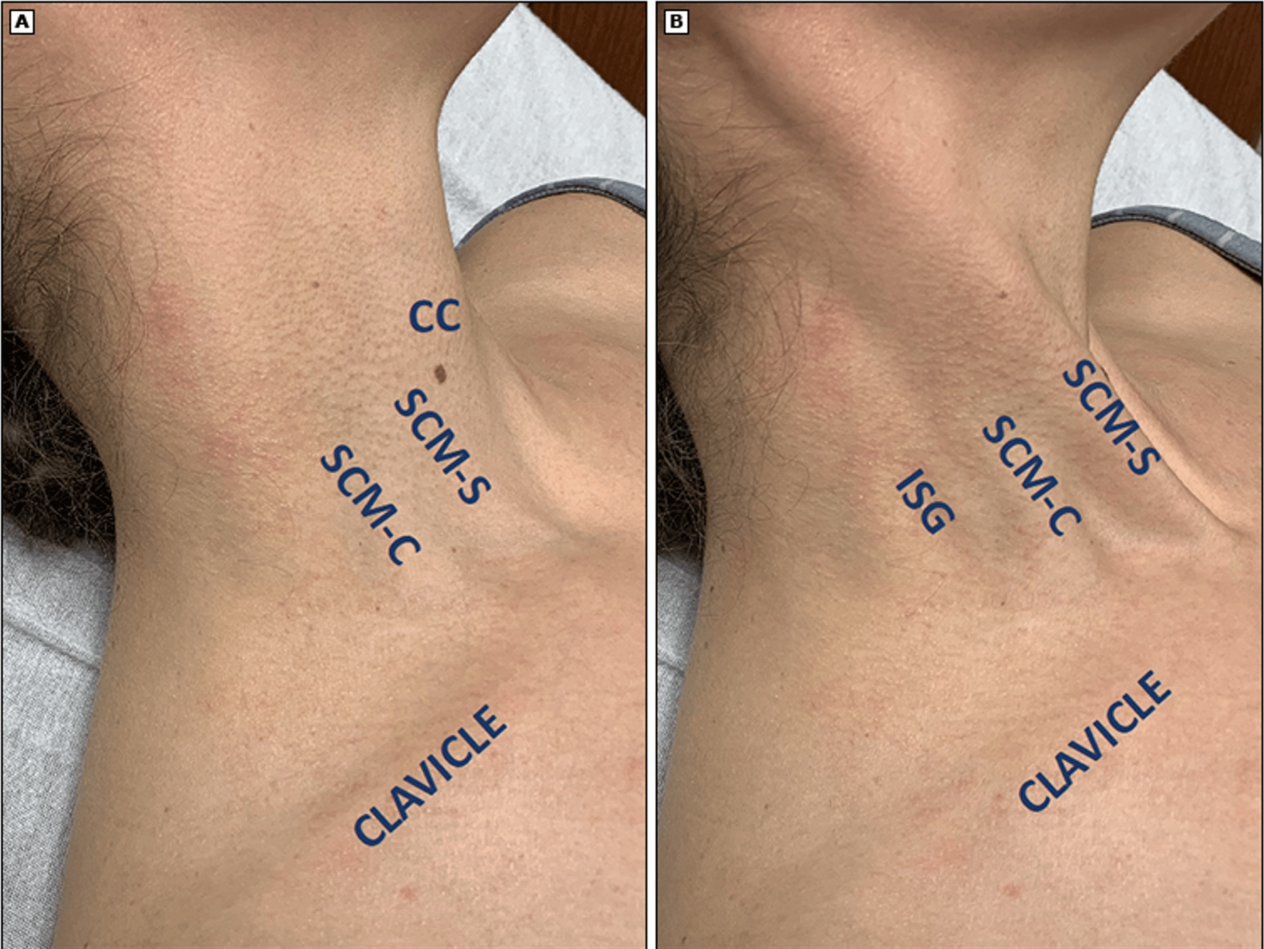
Anatomical Landmarks for ISB Administration (a) Surface landmarks used to perform an ISB. (b) The patient is flexing their neck to tense the SCM, which aids in identifying the posterior border of the SCM-C and the ISG. CC, cricoid cartilage; SCM-S, sternal head of sternocleidomastoid; SCM-C, clavicular head of sternocleidomastoid Image credit: Reproduced with permission from: Wilson EH, Klesius LL. Interscalene block procedure guide. In: UpToDate, Connor RF (Ed), Wolters Kluwer. Copyright © 2024 UpToDate, Inc. and/or its affiliates. All rights reserved [21].

**Figure 3:**
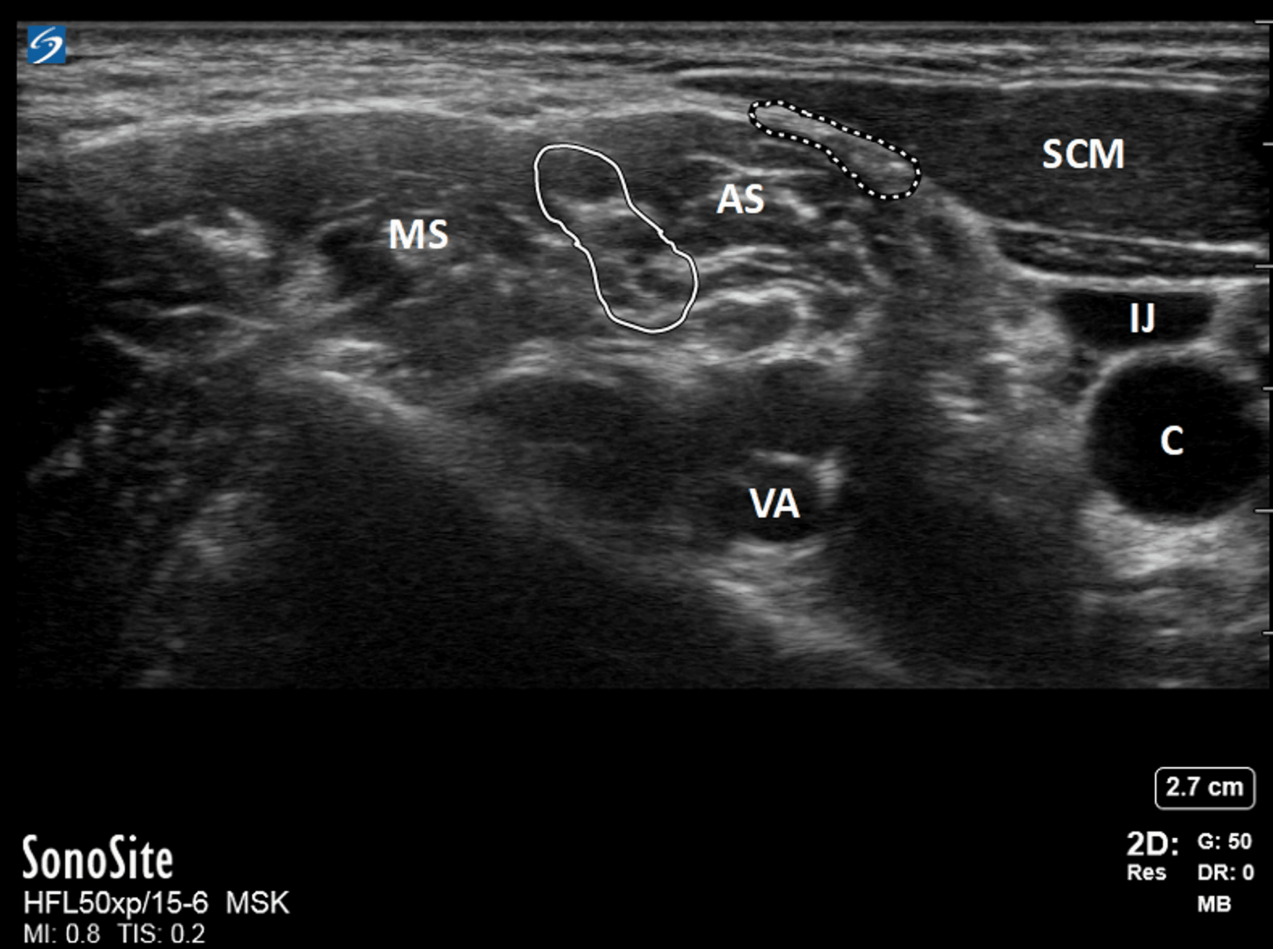
Ultrasound Image Showing the Brachial Plexus and Surrounding Anatomy at the ISG The solid white line highlights the brachial plexus, while the dashed white line indicates the superior cervical plexus. MS, middle scalene muscle; AS, anterior scalene muscle; IJ, internal jugular vein; VA, vertebral artery; CA, carotid artery Image credit: Reproduced with permission from: Wilson EH, Klesius LL. Interscalene block procedure guide. In: UpToDate, Connor RF (Ed), Wolters Kluwer. Copyright © 2024 UpToDate, Inc. and/or its affiliates. All rights reserved [21].

The ISG through which the brachial plexus trunks pass through can be palpated behind the clavicular head of the SCM (Figure 2). When using ultrasound, the plexus typically appears as two or three hollow circles ("stoplights") between the two scalenes, representing the superior, middle, and inferior trunks, although the inferior trunk may be harder to visualize as the muscle thickens (Figure 3). Once identified, an ISB is performed by injecting a local anesthetic, such as ropivacaine or bupivacaine, that blocks nerve impulses. Structures distal to the nerve block site subsequently experience sensory and motor function loss due to the blocked impulses [22]. The ISB is administered specifically at the level of the sixth cervical vertebra, the level at which the brachial plexus traverses through the ISG, targeting the C5 and C6 nerve roots, which form the superior trunk of the brachial plexus. This provides analgesia to the lateral two-thirds of the clavicle, proximal humerus, and the glenohumeral joint, making it an ideal choice for shoulder surgery analgesia, and hence why it is the most commonly utilized block and is recognized as the gold standard regional anesthetic technique for such procedures [11,17,23,24].

ISBs like other nerve blocks carry the risk of nerve injuries, such as neuropraxia or neurotmesis, due to inadvertent intraneural injection, local anesthetic systemic toxicity (LAST), hematoma formation, or direct trauma from the needle itself [25]. Complications of ISBs in particular include phrenic nerve palsy, given with subsequent hemidiaphragmatic paralysis, vertebral artery injury, Horner syndrome, subdural block, pneumothorax, and brachial plexus damage [23,26,27]. However, complications are extraordinarily rare, especially after the introduction of ultrasound-guided and nerve-stimulator-guided ISBs. Complications occur in around 2-3% of blocks, with permanent sequelae occurring in only about 0.25% of patients [28-34].

Comparison of CISB and single-shot ISB

Numerous studies have investigated the efficacy of continuous versus single-shot ISBs for post-operative pain management in shoulder surgery. These studies evaluate various aspects, including differences in pain relief, opioid consumption, recovery outcomes, and complication rates. This review includes studies from the last five years that specifically compared continuous and single-shot ISBs in any type of shoulder surgery. While standardization of techniques, such as ultrasound guidance, was not a criterion for inclusion, the diversity of methodologies provides a broader perspective on clinical outcomes. The following analysis provides a comprehensive comparison of these key factors, drawing on current evidence in the field (Table 1).

**Table 1:** Comparison of CISB and Single-Shot ISB

Aspect	Preferred Technique	Why?
Pain relief (<4 hrs)	Single-shot ISB	Single-shot ISB offers either comparable or enhanced pain relief compared to CISB [35-38]
Pain relief (24-48 hrs)	Mixed	Studies show mixed findings, with some indicating single-shot ISB as superior, others favoring CISB, and several reporting comparable pain relief between the two techniques [35-37,39-42]
Pain relief (>48 hrs)	CISB	CISB offers either comparable or enhanced pain relief compared to single-shot ISB [37,40,41,43]
Opioid use reduction	Mixed	Studies show mixed findings, with some indicating CISB as superior, but some favor single-shot ISB, and one reports comparable postoperative opioid usage between the two techniques [36,38,39,40,43,44]
Recovery and rehab	CISB	Although it may prolong hospital stay, CISB is superior in aiding recovery [37,39,44]
Complications	Single-shot ISB	Single-shot ISB is associated with fewer injection site complications and ED visits in most studies, but CISB is linked to fewer sleep disturbances and lower incidences of nausea and vomiting [35,38,39,41-44]
Procedural efficiency	Single-shot ISB	CISB requires more procedural time [37]

Technique

An ISB may be performed as either a single injection or with a continuous catheter. A single-shot ISB is relatively straightforward. A 22-gauge, short-bevel needle is advanced to the region described above via ultrasound guidance, and after confirming proper needle placement by aspiration, a single dose of local anesthetic, typically between 15 mL and 25 mL, is administered [21].

An interscalene catheter with a PCA pump is more complex. A larger, 17-18-gauge Tuohy needle with a curved tip is advanced to the region described above via ultrasound guidance, and after confirming proper needle placement by aspiration, a flexible catheter is threaded through the Tuohy needle and advanced beyond the needle tip. Next, the needle is removed, leaving the catheter in place, ensuring that it remains in the correct location near the brachial plexus. The catheter is then secured to the skin using an adhesive dressing, ensuring that it remains in place for continuous infusion. A bolus of local anesthetic is given through the catheter to establish the block, then a portable infusion pump is connected to the catheter to deliver a continuous infusion of local anesthetic over time. The infusion rate and volume depend on the desired duration and level of analgesia. The catheter is continually monitored for signs of displacement, leakage, or infection [21].

Pain Relief and Satisfaction

In comparing pain relief between single-shot ISB and CISB with a PCA pump for shoulder surgery, the literature reveals mixed results, with both techniques offering distinct advantages depending on the phase of recovery. Both methods have been shown to significantly reduce pain in the immediate postoperative period compared to control groups. For example, Wu (2002) with single-shot ISBs and Ilfeld (2003) with CISBs demonstrated significantly reduced postoperative pain in shoulder surgery patients who received an ISB versus those who did not [45,46]. 

When comparing single-shot ISB directly to CISB, studies show conflicting results. Weir (2020) found that shoulder arthroplasty patients receiving single-shot ISBs experienced less pain within the first four hours postoperatively compared to those receiving CISB. However, this early advantage of the single-shot technique diminished over time, with no significant differences in pain levels between the two groups from four to 36 hours postoperatively [37]. Other studies report no early differences between the two techniques at any time point. Kwater (2021) observed no discernible difference in pain scores during the first 48 hours after major shoulder surgery, suggesting that both techniques provide comparable pain relief during the early postoperative phase. Similarly, Teske (2022) found that single-shot ISBs offered noninferior analgesia compared to CISB for both arthroscopic and reconstructive shoulder surgeries, with similar pain scores on postoperative day one [35,42]. Other studies found no significant difference in pain between single-shot ISBs and CISBs on postoperative day 0, but a significant decrease in pain scores in the CISB group on postoperative day one [36,38]. 

As recovery progresses beyond the first day, the benefits of CISBs become more apparent in some studies. Uno (2024) determined a significant decrease in pain scores in patients receiving CISBs compared to single-shot ISBs on postoperative day two [37]. A meta-analysis by Vorobeichik (2018) found that CISB provided superior pain relief compared to single-shot ISB after 48 hours, prolonging the time to first analgesic use and improving patient satisfaction [43]. However, this later-recovery advantage of CISB over single-shot ISB is not supported by Hasan (2019) and Lee (2024), who both reported that CISBs significantly reduce pain compared to single-shot ISB on postoperative day one, but both studies also noted that the advantage of CISB faded after the first day, with pain levels becoming comparable between the two techniques [40,41]. Additionally, Uno (2024) also found no difference in pain between CISB and single-shot ISB groups on the third and fourth postoperative days [37]. 

Interestingly, some studies indicate that single-shot ISB may offer better pain control even beyond the immediate postoperative period. Levin (2022) reported that single-shot ISB patients had significantly lower mean pain scores at 24 and 36 hours postoperatively compared to those with CISB. Additionally, a smaller percentage of patients in the single-shot group experienced severe pain (pain scores of nine or 10), indicating that this technique may still offer effective analgesia even after the first day of surgery [39]. 

In summary, single-shot ISBs seem to offer better or equivalent pain relief in the immediate postoperative period, particularly within the first four hours. However, CISBs may provide superior pain relief beyond 24-48 hours, though this advantage may not always persist after the first day. Ultimately, the choice between single-shot and continuous blocks should depend on the specific clinical goals, the duration of expected pain, and patient preferences.

Opioid Consumption

The increasing burden of the opioid addiction crisis puts a strain on healthcare resources, increases treatment costs, and leads to lost productivity, which is costly to the U.S. economy. The CDC estimates that the opioid epidemic cost the U.S. $1.5 trillion in 2020 alone [47]. As such, it is vital to strive to find better techniques and procedures for pain management that reduce opioid requirements and decrease long-term use following surgery. It has been recently documented that the incidence of prolonged opioid use following arthroscopic shoulder procedures is approximately 8.3%, with higher rates observed among female patients and those with increased early postoperative opioid consumption [48]. Similarly, among opioid naïve patients who receive total shoulder arthroplasties, 38.5% required additional refills, 25.3% required an additional prescription, and 13.3% required prolonged opioid use six months after surgery [49]. 

For patients receiving either a single-shot ISB or CISB, opioids may still be indicated for supplementary pain management, but several studies have demonstrated that both single-shot ISBs and CISBs are associated with a significant reduction in opioid requirements compared to baseline analgesic methods following shoulder surgery [33,45,46]. Further studies have been conducted to compare the techniques and determine whether one reduces opioid requirements more effectively than the other.

Several studies support single-shot ISBs over CISBs in reducing opioid usage following shoulder surgery. Weir (2020) found that shoulder arthroplasty patients with single-shot ISBs consume fewer opioids overall and are discharged with fewer opioid prescriptions than CISBs [35]. Moreover, Levin (2022) saw that with shoulder arthroplasty, single-shot ISB recipients are more likely to remain opioid-free postoperatively, whereas patients receiving CISB are more likely to require opioid regimen escalation to PCA, indicating a greater reliance on opioids for pain control [39].

However, other studies conducted during the same period have yielded contrasting results. A meta-analysis by Vorobeichik (2018) demonstrated that CISBs lead to lower oral morphine consumption at both 24 and 48 hours postoperatively compared to single-shot ISBs [43]. Additionally, Yun (2021) found that compared to single-shot ISB, the CISB group received fewer opioids during the two days after surgery and that patients receiving CISBs experienced a significantly longer time to the first opioid request, suggesting more prolonged analgesic effects [44]. Hasan (2019) also saw substantially reduced opioid use during the first day postoperatively in CISB patients compared with single-shot ISB [40]. Finally, Bojaxhi (2019) demonstrated decreased opioid requirements in CISB patients compared to single-shot ISB patients on both postoperative days one and two [36]. 

In line with these contradictory findings, an additional study demonstrated no significant difference in opioid consumption between single-shot ISB and CISB within the first 48 hours after major shoulder surgery, indicating that both techniques provide comparable analgesia during this critical period [35]. This lack of significant difference in early opioid requirements suggests that despite the theoretical advantages of prolonged analgesia with CISB, patients who receive single-shot ISB do not experience a higher need for opioids. As such, both single-shot ISB and CISB appear to offer similar outcomes in terms of additional opioid therapy, and the choice between the two may ultimately depend on other factors such as the duration of the procedure, patient-specific needs, and provider preference. These findings emphasize that neither technique appears superior in reducing postoperative opioid consumption, highlighting the importance of individualized treatment strategies for optimizing pain management in shoulder surgeries.

Recovery, Motor Function, and Rehabilitation

When comparing single-shot ISBs and CISBs, several key differences emerge concerning recovery, motor function, and rehabilitation outcomes. Both single-shot and continuous ISBs have been shown to significantly shorten recovery room stays and achieve a greater early range of motion in shoulder surgery compared to control groups, facilitating quicker transitions to postoperative care [33,45]. 

While both techniques yielded similar quality recovery scores on the first postoperative day, the CISB group reported significantly improved recovery scores by postoperative day two, indicating a potential advantage in ongoing rehabilitation [44]. However, it is noteworthy that another study on shoulder arthroplasty patients found that the CISB cohort experienced a longer length of stay compared to the single-shot ISB group [39]. An additional study in shoulder arthroscopies found that CISBs are associated with greater shoulder extension at six months and one year postoperatively, and greater external rotation strength at six months postoperatively [37]. Overall, while both single-shot ISB and CISB facilitate early recovery, CISB may offer advantages in long-term rehabilitation and strength, though it could be associated with a longer hospital stay in certain cases.

Complications and Considerations

When comparing the complication rates and procedural efficiency of single-shot ISBs versus CISBs, the literature highlights a higher risk of complications and increased procedure time associated with CISBs. Kwater (2021) found that CISB not only required more time to complete the regional block procedure but also carried a higher incidence of complications compared to single-shot ISB [35]. This increase in time and complexity may be a consideration for clinicians seeking to balance effective pain management with procedural efficiency. Other studies similarly report an elevated risk of injection site complications and minor postoperative complications in CISB patients, reinforcing concerns about the safety of the continuous technique, particularly in the immediate postoperative period [41,42].

Additionally, shoulder surgery patients receiving CISB appeared to have more frequent emergency department visits in the weeks following surgery compared to those who received single-shot ISBs. Multiple studies observe that CISB patients had a higher rate of ED visits, further suggesting that the increased complexity and complications associated with continuous blocks may lead to additional postoperative issues. In contrast, single-shot ISB patients had fewer such revisits, which could make it a more attractive option in certain clinical scenarios where minimizing follow-up complications is a priority [38,39,42]. 

Despite the higher complication rates associated with CISB, the continuous technique does offer some benefits in postoperative recovery, particularly in reducing certain side effects. A meta-analysis by Vorobeichik (2018) found that CISB was more effective in reducing postoperative nausea and vomiting compared to single-shot ISBs, with no associated increase in complications [43]. Yun (2021) also highlighted that patients in the single-shot ISB group experienced a higher prevalence of sleep disturbances, nausea, and vomiting compared to those in the CISB group [44]. However, these findings may be a result of increased opioid use found in patients receiving single-shot ISBs in these two studies.

In summary, while CISB is associated with a higher rate of complications and increased procedural time, it may still be beneficial in reducing nausea and vomiting and improving patient recovery in some cases. Single-shot ISBs, on the other hand, appear to have fewer complications, require less time to administer, and result in fewer emergency department revisits, making it a more straightforward option for immediate postoperative pain control with fewer follow-up issues.

Clinical recommendations

The choice between single-shot ISBs and CISBs for shoulder surgery should be guided by the specific clinical goals, patient needs, and the expected course of recovery. Both techniques offer significant pain relief, but the timing of their effectiveness varies, with single-shot ISBs often providing superior analgesia in the immediate postoperative period, while CISBs may offer prolonged relief beyond 48 hours. While opioid consumption is reduced with both techniques, neither has emerged as consistently superior in minimizing opioid requirements, reinforcing the need for individualized treatment plans. In terms of recovery, CISBs may provide enhanced long-term rehabilitation outcomes, though they come with a higher incidence of complications and more frequent emergency department visits. Conversely, single-shot ISBs are associated with fewer complications, reduced ED visits, and faster procedural times, making them a more straightforward and efficient option in many scenarios.

Ultimately, both techniques present viable options for shoulder surgery pain management, with decisions hinging on the balance of short-term efficacy, complication risk, and patient-specific factors.

Future directions

Moving forward, as advancements in regional anesthesia continue, optimizing analgesic efficacy while minimizing complications is crucial. A few promising directions include the addition of dexamethasone to single-shot ISB and the exploration of alternatives such as suprascapular nerve blocks (SSNB) and axillary nerve blocks (ANB).

Recent studies suggest that adding perineural dexamethasone to single-shot ISBs can extend the duration of analgesia, addressing concerns that single-shot ISBs may not provide sufficient analgesia after postoperative day 0. Woo (2021) found that pain increase following ISB resolution was lower in the group receiving ISB with local anesthetic plus dexamethasone compared to the group receiving ISB with local anesthetic alone. Additionally, the incidence of rebound pain was lower in the dexamethasone group, and these patients experienced fewer sleep disturbances as well [50]. Moving on to comparisons between single-shot ISBs with dexamethasone and CISBs, one study observed that in patients who underwent arthroscopic rotator cuff repair, those who received a single-shot ISB with dexamethasone had superior pain relief at six hours postoperatively, and similar levels of pain thereafter, when compared to those who received a CISB [51]. Another study comparing single-shot ISBs with dexamethasone and CISBs found no significant differences in pain outcomes between the groups, highlighting the potential of single-shot ISBs with dexamethasone as a simpler alternative to CISBs [52]. This method is particularly attractive given the equivalence in analgesic duration without the added complexity and risks associated with catheter placement.

Overall, implementation of glucocorticoids in single-shot ISBs seems to provide meaningful benefits and may reduce the few downsides that come with it, while adding limited additional risk. Future research should focus on additional local compounds that can be administered alongside local anesthetics in ISBs. One such example is dexmedetomidine, a non-opioid sedative and analgesic agent that when injected locally, shows promise in potentiating the analgesic effects of ISBs and local dexamethasone in shoulder surgery patients [53].

SSNB and ANB are emerging as viable alternatives to ISB, particularly for shoulder surgery patients at high risk of complications such as those with chronic obstructive pulmonary disease or obstructive sleep apnea. While ISB is highly effective for immediate postoperative pain control, it can precipitate complications, including respiratory dysfunction, Horner’s syndrome, and hoarseness of voice. SSNB and ANB, on the other hand, are posited to offer comparable analgesia with a significantly lower incidence of these block-associated complications. A systematic review comparing the efficacies and complication rates of SSNBs and ISBs found that ISBs provided superior pain relief immediately postoperatively but did not impact opioid consumption in this same timeframe. Additionally, pain scores and opioid usage between the two groups were similar after the six-hour mark postoperatively. While there was no difference in respiratory events post-block, SSNBs were associated with a lower incidence of respiratory function impairment, Horner’s syndrome, and voice hoarseness [54]. A meta-analysis comparing SSNBs and ISBs in shoulder arthroscopy found ISBs producing improved pain relief in the first hour after surgery, but SSNBs produced similar relief in the following six hours and even superior relief at the 12-hour postoperative mark. On postoperative days one and two, pain scores were similar between the two groups. There was no difference between the groups in opioid usage, duration of stay, patient satisfaction, vomiting, and local tenderness. However, the SSNB group had lower rates of numbness, Horner’s syndrome, respiratory function impairment, and hoarseness [55].

Another meta-analysis by Sun (2021) comparing a combination of SSNB/ANB versus ISB in shoulder arthroscopies demonstrated that ISB provided better relief for the first six hours postoperatively, but SSNB plus ANB provided similar levels of pain relief following this period. SSNB plus ANB had a lower incidence of inadvertent numbness, weakness, Horner’s syndrome, and respiratory function impairment but had similar rates of hoarseness and nausea/vomiting. Additionally, patient-reported satisfaction was comparable between the two groups [56].

Alternative regional nerve blocks such as SSNB and ANB may be a suitable analgesic option for shoulder surgery, potentially offering fewer complications compared to the ISB. These studies only looked at outcomes in shoulder arthroscopy patients, leaving out other more invasive and pain-ridden shoulder surgeries, such as arthroplasty, which may prove to have differing outcomes. Thus, more research is needed to determine whether these positive findings can be extended to shoulder arthroplasties and other more complex cases.

## Conclusions

In this review, single-shot and continuous catheter ISBs were evaluated for postoperative pain management in shoulder surgery. Single-shot ISBs were found to provide efficient immediate pain relief with fewer complications and quicker procedural times, making them suitable for patients prioritizing simplicity and safety. Conversely, CISBs offered prolonged analgesia, reduced opioid consumption, and enhanced recovery outcomes but were associated with higher risks and greater procedural complexity. Emerging alternatives, such as perineural dexamethasone adjuncts and suprascapular or axillary nerve blocks, show the potential to optimize pain relief while minimizing risks. These findings underscore the importance of tailoring anesthesia techniques to individual patient needs and balancing recovery goals with complication risks to enhance postoperative care.

## References

[1] Wagner ER, Farley KX, Higgins I, Wilson JM, Daly CA, Gottschalk MB (2020). The incidence of shoulder arthroplasty: rise and future projections compared with hip and knee arthroplasty. J Shoulder Elbow Surg.

[2] Jain NB, Higgins LD, Losina E, Collins J, Blazar PE, Katz JN (2014). Epidemiology of musculoskeletal upper extremity ambulatory surgery in the United States. BMC Musculoskelet Disord.

[3] Bullingham RE (1984). Postoperative pain. Postgrad Med J.

[4] Weber SC, Jain R, Parise C (2007). Pain scores in the management of postoperative pain in shoulder surgery. Arthroscopy.

[5] Ndebea AS, van den Heuvel SA, Temu R, Kaino MM, van Boekel RL, Steegers MA (2020). Prevalence and risk factors for acute postoperative pain after elective orthopedic and general surgery at a tertiary referral hospital in Tanzania. J Pain Res.

[6] Arefayne NR, Tegegne SS, Gebregzi AH, Mustofa SY (2020). Incidence and associated factors of post-operative pain after emergency orthopedic surgery: a multi-centered prospective observational cohort study. Int J Surg Open.

[7] Gerbershagen HJ, Aduckathil S, van Wijck AJ, Peelen LM, Kalkman CJ, Meissner W (2013). Pain intensity on the first day after surgery: a prospective cohort study comparing 179 surgical procedures. Anesthesiology.

[8] Lindberg MF, Grov EK, Gay CL, Rustøen T, Granheim TI, Amlie E, Lerdal A (2013). Pain characteristics and self-rated health after elective orthopaedic surgery - a cross-sectional survey. J Clin Nurs.

[9] Xiao M, Cohen SA, Cheung EV, Freehill MT, Abrams GD (2021). Pain management in shoulder arthroplasty: a systematic review and network meta-analysis of randomized controlled trials. J Shoulder Elbow Surg.

[10] Gabriel RA, Nagrebetsky A, Kaye AD, Dutton RP, Urman RD (2016). The patterns of utilization of interscalene nerve blocks for total shoulder arthroplasty. Anesth Analg.

[11] Sripada R, Bowens C Jr (2012). Regional anesthesia procedures for shoulder and upper arm surgery upper extremity update--2005 to present. Int Anesthesiol Clin.

[12] Al-Kaisy A, McGuire G, Chan VW, Bruin G, Peng P, Miniaci A, Perlas A (1998). Analgesic effect of interscalene block using low-dose bupivacaine for outpatient arthroscopic shoulder surgery. Reg Anesth Pain Med.

[13] Hadzic A, Williams BA, Karaca PE (2005). For outpatient rotator cuff surgery, nerve block anesthesia provides superior same-day recovery over general anesthesia. Anesthesiology.

[14] Codding JL, Getz CL (2018). Pain management strategies in shoulder arthroplasty. Orthop Clin North Am.

[15] Leafblad N, Asghar E, Tashjian RZ (2022). Innovations in shoulder arthroplasty. J Clin Med.

[16] Bayot ML, Nassereddin A, Varacallo M (2023). Anatomy, Shoulder and Upper Limb, Brachial Plexus. Anatomy, Shoulder and Upper Limb, Brachial Plexus.

[17] Borgeat A, Ekatodramis G (2002). Anaesthesia for shoulder surgery. Best Pract Res Clin Anaesthesiol.

[18] Basta M, Sanganeria T, Varacallo M (2022). Anatomy, Shoulder and Upper Limb, Suprascapular Nerve. Anatomy, Shoulder and Upper Limb, Suprascapular Nerve.

[19] Okwumabua E, Thompson JH (2023). Anatomy, Shoulder and Upper Limb, Axillary Nerve. Anatomy, Shoulder and Upper Limb, Axillary Nerve.

[20] Bordoni B, Varacallo M (2023). Anatomy, Head and Neck, Scalenus Muscle. Anatomy, Head and Neck, Scalenus Muscle.

[21] (2024). Interscalene block procedure guide. https://www.uptodate.com/contents/interscalene-block-procedure-guide.

[22] Zisquit J, Nedeff N (2022). Interscalene Block. Interscalene Block.

[23] Fredrickson MJ, Krishnan S, Chen CY (2010). Postoperative analgesia for shoulder surgery: a critical appraisal and review of current techniques. Anaesthesia.

[24] Warrender WJ, Syed UA, Hammoud S, Emper W, Ciccotti MG, Abboud JA, Freedman KB (2017). Pain management after outpatient shoulder arthroscopy: a systematic review of randomized controlled trials. Am J Sports Med.

[25] Pester JM, Hendrix JM, Varacallo M (2023). Brachial Plexus Block Techniques. Brachial Plexus Block Techniques.

[26] Liu Z, Li YB, Wang JH, Wu GH, Shi PC (2022). Efficacy and adverse effects of peripheral nerve blocks and local infiltration anesthesia after arthroscopic shoulder surgery: a Bayesian network meta-analysis. Front Med (Lausanne).

[27] Lenters TR, Davies J, Matsen FA 3rd (2007). The types and severity of complications associated with interscalene brachial plexus block anesthesia: local and national evidence. J Shoulder Elbow Surg.

[28] Singh A, Kelly C, O'Brien T, Wilson J, Warner JJ (2012). Ultrasound-guided interscalene block anesthesia for shoulder arthroscopy: a prospective study of 1319 patients. J Bone Joint Surg Am.

[29] Takayama K, Shiode H, Ito H (2022). Ultrasound-guided interscalene block anesthesia performed by an orthopedic surgeon: a study of 1322 cases of shoulder surgery. JSES Int.

[30] Lim JA, Sung SY, Lee JH, Lee SY, Kwak SG, Ryu T, Roh WS (2020). Comparison of ultrasound-guided and nerve stimulator-guided interscalene blocks as a sole anesthesia in shoulder arthroscopic rotator cuff repair: a retrospective study. Medicine (Baltimore).

[31] Kapral S, Greher M, Huber G, Willschke H, Kettner S, Kdolsky R, Marhofer P (2008). Ultrasonographic guidance improves the success rate of interscalene brachial plexus blockade. Reg Anesth Pain Med.

[32] Ghodki PS, Singh ND (2016). Incidence of hemidiaphragmatic paresis after peripheral nerve stimulator versus ultrasound guided interscalene brachial plexus block. J Anaesthesiol Clin Pharmacol.

[33] Liu SS, Zayas VM, Gordon MA (2009). A prospective, randomized, controlled trial comparing ultrasound versus nerve stimulator guidance for interscalene block for ambulatory shoulder surgery for postoperative neurological symptoms. Anesth Analg.

[34] Girdler-Hardy TP, Webb C, Menon G (2015). Improved safety and efficacy of ultrasound-guided interscalene nerve block vs a nerve-stimulator guided technique. Br J Anaesth.

[35] Weir TB, Simpson N, Aneizi A (2020). Single-shot liposomal bupivacaine interscalene block versus continuous interscalene catheter in total shoulder arthroplasty: opioid administration, pain scores, and complications. J Orthop.

[36] Kwater AP, Hernandez N, Artime C, de Haan JB (2021). Interscalene block for analgesia in orthopedic treatment of shoulder trauma: single-dose liposomal bupivacaine versus perineural catheter. Local Reg Anesth.

[37] Bojaxhi E, Lumermann LA, Mazer LS, Howe BL, Ortiguera CJ, Clendenen SR (2019). Interscalene brachial plexus catheter versus single-shot interscalene block with periarticular local infiltration analgesia for shoulder arthroplasty. Minerva Anestesiol.

[38] Uno T, Mura N, Yuki I, Oishi R, Takagi M (2024). The effect of continuous interscalene brachial plexus block for arthroscopic rotator cuff repair. Asia Pac J Sports Med Arthrosc Rehabil Technol.

[39] Teske LG, Pill SG, Lutz A (2022). Single shot interscalene regional anesthesia provides noninferior analgesia and decreased complications compared with an indwelling catheter for arthroscopic and reconstructive shoulder surgery. J Shoulder Elbow Surg.

[40] Levin JM, Charalambous LT, Girden A (2022). Interscalene block with liposomal bupivacaine versus continuous interscalene catheter in primary total shoulder arthroplasty. J Shoulder Elbow Surg.

[41] Hasan SS, Rolf RH, Sympson AN, Eten K, Elsass TR (2019). Single-shot versus continuous interscalene block for postoperative pain control after shoulder arthroplasty: a prospective randomized clinical trial. J Am Acad Orthop Surg Glob Res Rev.

[42] Lee JY, Wu JC, Chatterji R (2024). Complication rates and efficacy of single-injection vs. continuous interscalene nerve block: a prospective evaluation following arthroscopic primary rotator cuff repair without a concomitant open procedure. JSES Int.

[43] Vorobeichik L, Brull R, Bowry R, Laffey JG, Abdallah FW (2018). Should continuous rather than single-injection interscalene block be routinely offered for major shoulder surgery? A meta-analysis of the analgesic and side-effects profiles. Br J Anaesth.

[44] Yun S, Jo Y, Sim S (2021). Comparison of continuous and single interscalene block for quality of recovery score following arthroscopic rotator cuff repair. J Orthop Surg (Hong Kong).

[45] Wu CL, Rouse LM, Chen JM, Miller RJ (2002). Comparison of postoperative pain in patients receiving interscalene block or general anesthesia for shoulder surgery. Orthopedics.

[46] Ilfeld BM, Morey TE, Wright TW, Chidgey LK, Enneking FK (2003). Continuous interscalene brachial plexus block for postoperative pain control at home: a randomized, double-blinded, placebo-controlled study. Anesth Analg.

[47] (2024). The economic toll of the opioid crisis reached nearly $1.5 trillion in 2020. https://www.jec.senate.gov/public/index.cfm/democrats/2022/9/the-economic-toll-of-the-opioid-crisis-reached-nearly-1-5-trillion-in-2020.

[48] Gil JA, Gunaseelan V, DeFroda SF, Brummett CM, Bedi A, Waljee JF (2019). Risk of prolonged opioid use among opioid-naïve patients after common shoulder arthroscopy procedures. Am J Sports Med.

[49] Spencer CC, Pflederer JA, Wilson JM, Dawes AM, Gottschalk MB, Wagner ER (2021). Opioid use following a total shoulder arthroplasty: who requires refills and for how long?. JSES Int.

[50] Woo JH, Lee HJ, Oh HW, Lee JW, Baik HJ, Kim YJ (2021). Perineural dexamethasone reduces rebound pain after ropivacaine single injection interscalene block for arthroscopic shoulder surgery: a randomized controlled trial. Reg Anesth Pain Med.

[51] Kim YS, Park Y, Koh HJ (2022). Is there a difference between perineural dexamethasone with single-shot interscalene block (SSIB) and interscalene indwelling catheter analgesia (IICA) for early pain after arthroscopic rotator cuff repair? A pilot study. J Clin Med.

[52] Pai BHP, Bohaczuk S, Jinadu S (2024). Comparison of analgesic efficacy of continuous perineural catheter, liposomal bupivacaine, and dexamethasone as an adjuvant for interscalene block in total shoulder arthroplasty: a triple-blinded randomized controlled trial. J Shoulder Elbow Surg.

[53] Wei XM, Liu Z, Lv LC, Wu GH, Sun PY, Gu CP, Shi PC (2023). Comparison of dexmedetomidine and dexamethasone as adjuvants to the ultrasound-guided interscalene nerve block in arthroscopic shoulder surgery: a systematic review and Bayesian network meta-analysis of randomized controlled trials. Front Med (Lausanne).

[54] White L, Reardon D, Davis K, Velli G, Bright M (2022). Anterior suprascapular nerve block versus interscalene brachial plexus block for arthroscopic shoulder surgery: a systematic review and meta-analysis of randomized controlled trials. J Anesth.

[55] Sun C, Ji X, Zhang X, Ma Q, Yu P, Cai X, Yang H (2021). Suprascapular nerve block is a clinically attractive alternative to interscalene nerve block during arthroscopic shoulder surgery: a meta-analysis of randomized controlled trials. J Orthop Surg Res.

[56] Sun C, Zhang X, Ji X, Yu P, Cai X, Yang H (2021). Suprascapular nerve block and axillary nerve block versus interscalene nerve block for arthroscopic shoulder surgery: a meta-analysis of randomized controlled trials. Medicine (Baltimore).

